# DICER1-Related Pediatric Thyroid Neoplasm with Follicular and Morular Growth: A Tumor that Did Not Read the Textbook

**DOI:** 10.1007/s12022-025-09874-z

**Published:** 2025-09-02

**Authors:** José Manuel Cameselle-Teijeiro, Sangeeta Verma, Anthony Penn, Chitra Sethuraman, Isabel Amendoeira, Pablo Garrido-Gil, José Luís Labandeira-García, Beatriz Sobrino, Clara Ruíz-Ponte, Manuel Sobrinho-Simões

**Affiliations:** 1 Department of Pathology, Clinical University Hospital of Santiago de Compostela, Galician Healthcare Service (SERGAS), Health Research Institute of Santiago de Compostela (IDIS), Medical Faculty, University of Santiago de Compostela, Travesía Choupana s/n, Santiago de Compostela, 15706 Spain; 2 Department of Histopathology, The Christie NHS Foundation Trust, Manchester, UK; 3 Department of Paediatric Oncology, Royal Manchester Children’s Hospital, Manchester, UK; 4 Department of Paediatric Histopathology, Manchester University NHS Foundation Trust, Oxford Road, Manchester, UK; 5 Department of Pathology, ULS São João, Institute of Molecular Pathology and Immunology (IPATIMUP), i3S-Institute for Research & Innovation in Health, University of Porto, Porto, Portugal; 6 Research Center for Molecular Medicine and Chronic Diseases (CiMUS), Networking Research Center on Neurodegenerative Diseases (CIBERNED), Health Research Institute of Santiago de Compostela (IDIS), University of Santiago de Compostela, Santiago de Compostela, Spain; 7 Fundación Pública Galega de Medicina Xenómica, Centro de Investigación Biomédica en Red de Enfermedades Raras (CIBERER), Servicio Galego de Saúde (SERGAS), Instituto de Investigación Sanitaria de Santiago de Compostela (IDIS), Grupo de Medicina Xenómica-Universidade de Santiago de Compostela, Santiago de Compostela, Spain; 8 Department of Pathology, Medical Faculty, Institute of Molecular Pathology and Immunology (IPATIMUP), i3S-Institute for Research & Innovation in Health, University of Porto, Porto, 4200-135 Portugal

**Keywords:** Thyroid, *DICER1*, *CTNNB1*, Pediatric thyroid cancer, Thyroid tumor, Morular structures, β-Catenin

## Abstract

Thyroid lesions associated with *DICER1* syndrome include multifocal hyperplastic and benign neoplastic proliferations (follicular nodular disease) with characteristic macrofollicular and/or intrafollicular centripetal papillary growth patterns, frequently associated with atrophic and involutional changes. There are also well-differentiated thyroid carcinomas showing intermediate-type nuclei, sometimes combining high-grade areas (tumor-in-tumor pattern) and poorly differentiated carcinomas. Here, for the first time, we describe an encapsulated follicular cell thyroid tumor showing a mixed follicular and morular growth pattern, which presented in an 11-year-old girl with follicular nodular disease and a constitutional (germline) *DICER1* p.(Tyr1357fs*18) pathogenic variant. The tumoral follicular component showed colloid and tumor cells with round nuclei, frequent chromatin clearing, and overlapping without grooves or pseudoinclusions (intermediate-type nuclei). There were scattered mitotic figures, but no tumor necrosis, infiltration, or vascular invasion. The morular structures lacked keratinization. The follicular areas were positive for TTF1/NKX2, PAX8, thyroglobulin, thyroperoxidase, keratin clones CKAE1/AE3 and 34bE12, CK19, and vimentin, whereas the morular component was positive for CKAE1/AE3, CK19, CD10, and CDX2. Aberrant (nuclear and cytoplasmic) immunolabeling pattern for β-catenin was limited to the morular structures. The Ki67 proliferation index was 21% in the follicular component and less than 1% in the morulae. In addition to the constitutional *DICER1* p.(Tyr1357fs*18) variant, the somatic *DICER1* p.(Asp1910Tyr) oncogenic variant and the somatic *CTNNB1* p.(Thr41Ala) oncogenic variant were also identified in this tumor. This “DICER1-related pediatric thyroid neoplasm with follicular and morular growth” expands the spectrum of *DICER1*-associated thyroid lesions. Indirectly, the absence of follicular markers only in the areas with WNT/β-catenin pathway activation (morular structures) in this neoplasm could explain the absence of follicular differentiation in cribriform morular thyroid carcinoma. The additional study of one of the accompanying thyroid nodules (follicular nodular disease) confirmed the constitutional *DICER1* variant, along with *DICER1* p.(Asp1709Gly) and p.(Asp1810Val) variants.

## Introduction

*DICER1* syndrome is associated with a wide range of characteristic tumors and dysplastic disorders caused by heterozygous germline pathogenic variants in the *DICER1* gene [1]. *DICER1*, located on chromosome 14q32, functions canonically as an RNase III endoribonuclease essential for microRNA biogenesis. RNase IIIb-mutated *DICER1* exhibits abnormal 5p microRNA processing, resulting in an altered 5p:3p microRNA ratio, which, together with simultaneous dysregulation of gene expression, underlies tumor development [2, 3]. Within the histological spectrum of *DICER1*-associated tumors, thyroid follicular nodular disease (TFND) is one of the most prevalent lesions [4]. In addition to TFND, differentiated thyroid carcinoma [1, 5] and poorly differentiated thyroid carcinoma [6, 7] have been described in childhood or adolescence related to germline *DICER1* mutations, while thyroblastoma is more common in adults and is typically associated with somatic mutations in *DICER1* [8]. In the context of *DICER1* syndrome, thyroid involvement often also includes a series of histopathological features such as follicular adenomas with macrofollicular growth pattern and papillary infoldings [9], atrophic and/or involutional changes [10, 11], and the presence of intermediate nuclei, which may alert the pathologist to the possibility of *DICER1* syndrome [9, 12, 13].

Here, we describe, for the first time, an encapsulated follicular cell thyroid tumor showing a mixed follicular and morular growth pattern, presented in a girl with a germline *DICER1* gene mutation. This tumor broadens the spectrum of *DICER1*-associated thyroid tumors. The morphological, immunohistochemical, and molecular characteristics of this DICER1-related pediatric thyroid neoplasm with follicular and morular growth also provide additional information on the pathogenesis of both pediatric thyroid tumors and cribriform morular thyroid carcinoma [14].

## Material and Methods

### Case History

An 11-year-old girl underwent a right lobectomy to complete a thyroidectomy for multiple thyroid nodules. The patient had been identified as having *DICER1* syndrome after genetic screening following the diagnosis of the syndrome in her older sister who was treated for pineoblastoma. After follow-up ultrasound studies, a left hemithyroidectomy had been performed, leading to the pathological diagnosis of “Dominant hyperplastic nodule of a syndromic situation that may be associated with *DICER1* mutation.” Nine months after the first surgery, the thyroidectomy was completed due to several enlarged nodules in the remaining thyroid lobe. Based on the lack of invasiveness of the tumor, radioactive iodine was not administered. The patient receives hormone replacement treatment, is being monitored periodically (including serum thyroglobulin levels), and is doing well with no evidence of other alterations, 17 months after the second surgical intervention.

### Pathological and Immunohistochemical Analysis

The right hemithyroidectomy specimen was fixed in neutral, phosphate-buffered, 10% formalin and included in paraffin blocks. Formalin-fixed paraffin-embedded (FFPE) tissue sections were stained with hematoxylin–eosin; immunohistochemical studies were also performed on 4-µm-thick paraffin sections using a peroxidase-conjugated-labeled dextran polymer (Dako EnVision Peroxidase/DAB; Dako, Glostrup, Denmark), with 3,3′-diaminobenzidine as the chromogen, and using a series of primary antibodies whose main characteristics are described in Table 1.

**Table 1:** Immunohistochemical characteristics of the DICER1-related pediatric thyroid neoplasm with follicular and morular growth

Antibodies	Clone	Dilution	Antigen retrieval	Manufacturer	Tumor cells	
					Follicular component	Morular structures
TG	Polyclonal	Ready to use	pH 6	Dako, Glostrup, Denmark	+ +	-
TPO	MoAb47	1/50	pH 9	Dako	+ +	-
CT	Polyclonal	1/400		Dako	-	-
TTF1	SPT24	1/100	pH 9	Gennova, Sevilla, Spain	+ +	-/+ *
PAX8	SP348	1/100	pH 9	Gennova	+ +	-/+ *
β-catenin	β-Catenin-1	Ready to use	pH 9	Dako	m	mcn
Keratins	CKAE1/AE3	Ready to use	pH 9	Dako	+ +	+ +
Keratins	CK19	Ready to use	pH 9	Dako	+	+ +
Keratins	34bE12	Ready to use	pH 9	Dako	-	-
Vimentin	V9	Ready to use	pH 9	Dako	+	-
CD5	4C7	Ready to use	pH 9	Dako	-	-
CD10	DAK-CD10	Ready to use	pH 9	Dako	-*	+ +
CDX2	DAK-CDX2	Ready to use	pH 9	Dako	-	+
ER	EP1/IR044IVD	Ready to use	pH 9	Dako	-	-
PR	636/IR068	Ready to use	pH 9	Dako	-	-
p40	DAK-p40	Ready to use	pH 9	Dako	-	-
SALL4	IHC659	1:50	pH 9	GenomeMe, BC, Canada	-	-
Ki67	MIB1	1:200	pH 9	Dako	21%	< 1%

*TG*, thyroglobulin; *TPO*, thyroperoxidase; *CT*, calcitonin; *TTF1/NKX2‐1*, NK2 homeobox 1; *PAX8*, paired box 8; *ER*, estrogen receptors-α; *PR*, progesterone receptors; *m*, membrane positivity; *mcn*, membrane and cytoplasm positivity with strong nuclear positivity. *Positivity in a few isolated cells

### Laser Capture Microdissection

Laser capture microdissection (LCM) was used to isolate pools of thyroid cells from (1) main nodule, including both follicular and morular pattern areas, (2) morular structures, (3) one of the macrofollicular thyroid adenomas, and (4) “normal” thyroid tissue, from FFPE tissue sections stained with hematoxylin. LCM was performed using a PALM MicroBeam system (Zeiss, Jena, Germany), equipped with an inverted microscope (Axio Observer.Z1), a motorized stage, and an ultraviolet laser, and controlled by PALM RoboSoftware version 4.2 (Zeiss) (see details in Garrido-Gil et al. [15]). The mixed follicular and morular areas of the main nodule and the other cell pools were identified and visualized under bright-field microscopy using a 40 × objective, with the aid of an AxioCam Icc camera (Zeiss). The different cell pools were selected and outlined using the software interface, and subsequently microdissected and catapulted into Zeiss adhesive microtube caps via laser pulses. Captured cell pools were lysed and stored at − 80 °C until further processing.

### Genetic Analysis

Whole-exome sequencing (WES) was carried out on DNA extracted from different pools of FFPE tissues obtained by LCM. DNA extraction was undertaken using the QIAamp DNA FFPE Advanced kit (Qiagen, Germany). WES was performed using the KAPA HyperPrep Kit (Roche Sequencing Solutions, Inc.) for library preparation and KAPA HyperExome v2 (Roche) for capturing the region of interest following KAPA HyperCap Workflow manufacturer’s protocol. Exome libraries were sequenced on the NovaSeq 6000 (Illumina, Inc.) with 2 × 100 bp pair-end reads. The sequence reads were aligned to the human reference genome GRCh38 with the Illumina software DRAGEN v4.3.13, with an average coverage greater than 150 ×. To obtain the tumor-exclusive variants, the normal thyroid tissue was used for the normal-tumor paired analyses. The exome analysis was restricted to the *APC* (NM_000038.6), *AXIN1* (NM_003502.4), *AXIN2* (NM_004655.4), and *CTNNB1* (NM_001904.4) genes involved in the canonical WNT/β-catenin pathway, along with *DICER1* (NM_177438.3) and other genes involved in the pathogenesis of follicular thyroid tumors: *BRAF* (NM_004333.6), *NRAS* (NM_002524.5), *KRAS* (NM_033360.4), *HRAS* (NM_176795.5), *EIF1AX* (NM_001412.4), *PTEN* (NM_000314.8), *TERT* (NM_198253.3), *PI3KCA* (NM_006218.4), *KMT2C* (NM_170606.3), *KMT2D* (NM_003482.4), *CHEK2* (NM_007194.4), *ATM* (NM_000051.4), and *TP53* (NM_000546.6). Genetic variants were described according to the Human Genome Variation Society (HGVS) (https://varnomen.hgvs.org/), and the classification of pathogenicity was performed according to the standards of pathogenicity of somatic variants in cancer [16].

## Results

### Pathological and Immunohistochemical Features

**Fig. 1:**
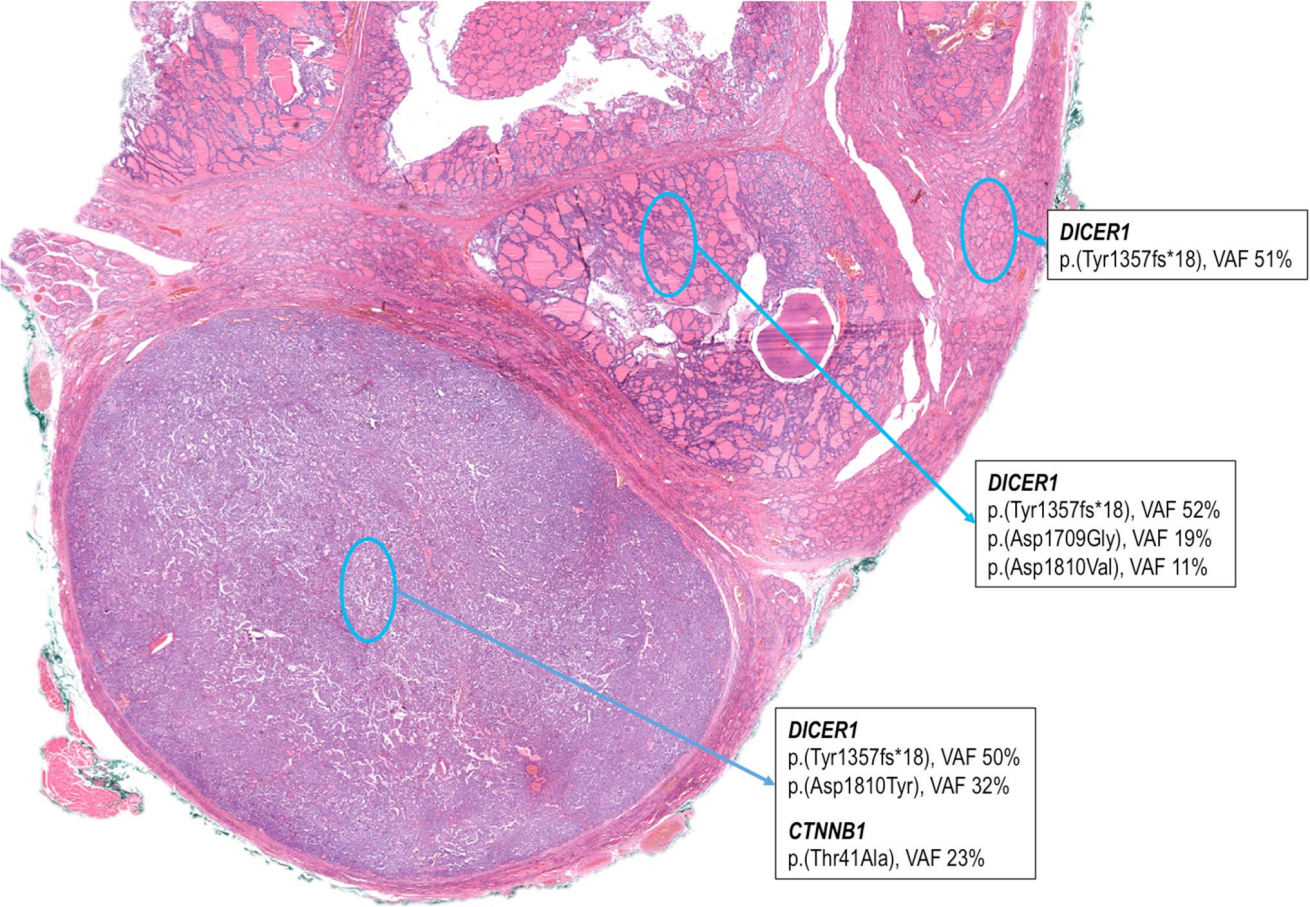
DICER1-related pediatric thyroid neoplasm with follicular and morular growth. Low magnification of a thyroid section showing various nodules (top) and the tumor with follicular and morular growth (bottom). The same constitutional *DICER1* p.(Tyr1357fs*18) mutation identified in the histologically normal thyroid tissue (right) was also confirmed in one adenomatous nodule and in the tumor with follicular and morular growth (bottom). Additionally, two different *DICER1* p.(Asp1709Gly) and p.(Asp1810Val) mutations were detected in the adenomatous nodule. The tumor with follicular and morular growth showed another different *DICER1* p.(Asp1810Tyr) mutation along with a *CTNNB1* p.(Thr41Ala) mutation

**Fig. 2:**
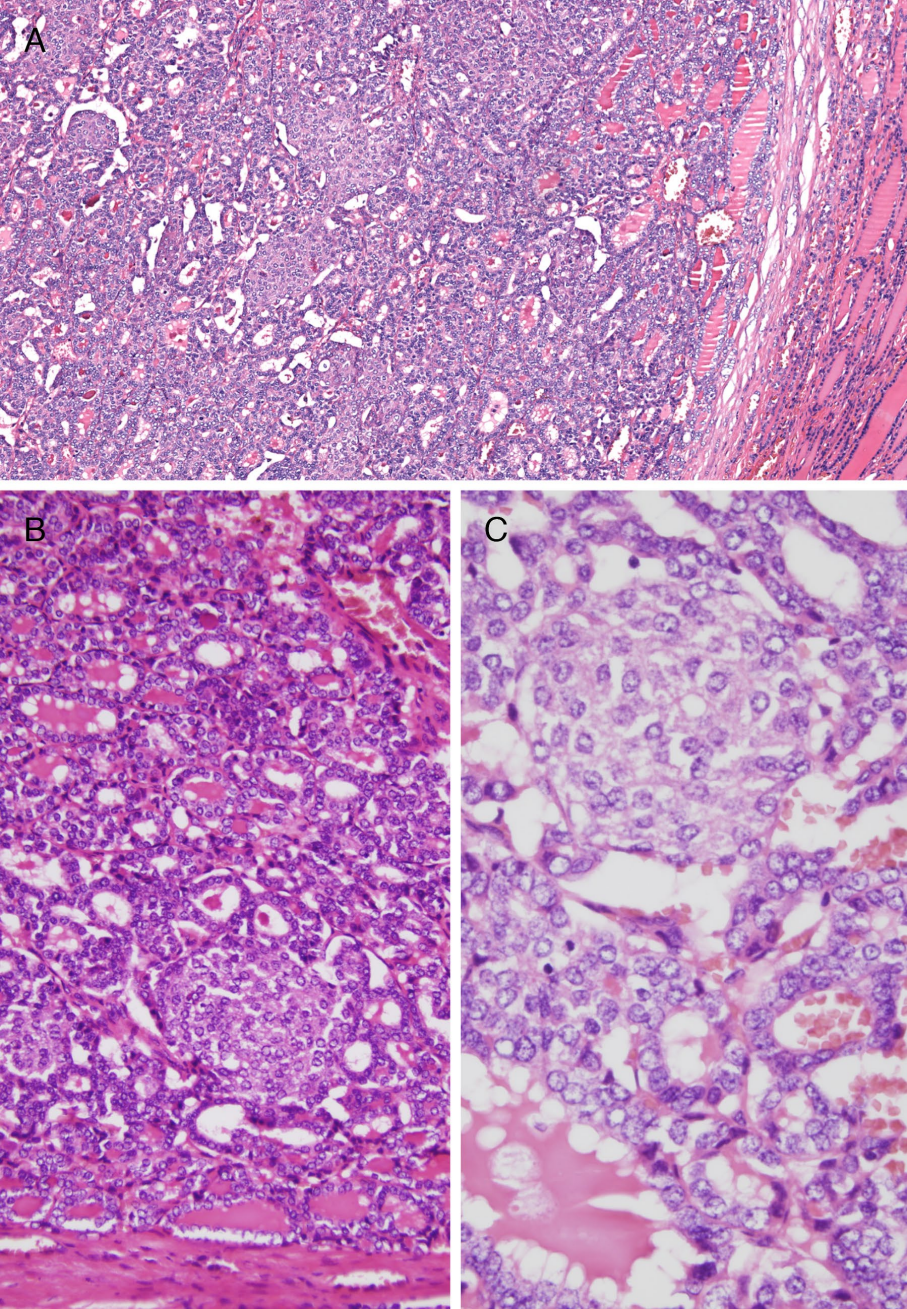
DICER1-related pediatric thyroid neoplasm with follicular and morular growth. The tumor is well-delimited by a thin fibrous capsule (**A**) and composed of a mixed combination of follicles and morular structures (**A**–**C**). The follicular pattern is predominant, usually containing colloid and lined by cells showing nuclear overlapping and clear chromatin (**B, C**). Morular structures lack keratinization (**C**)

**Fig. 3:**
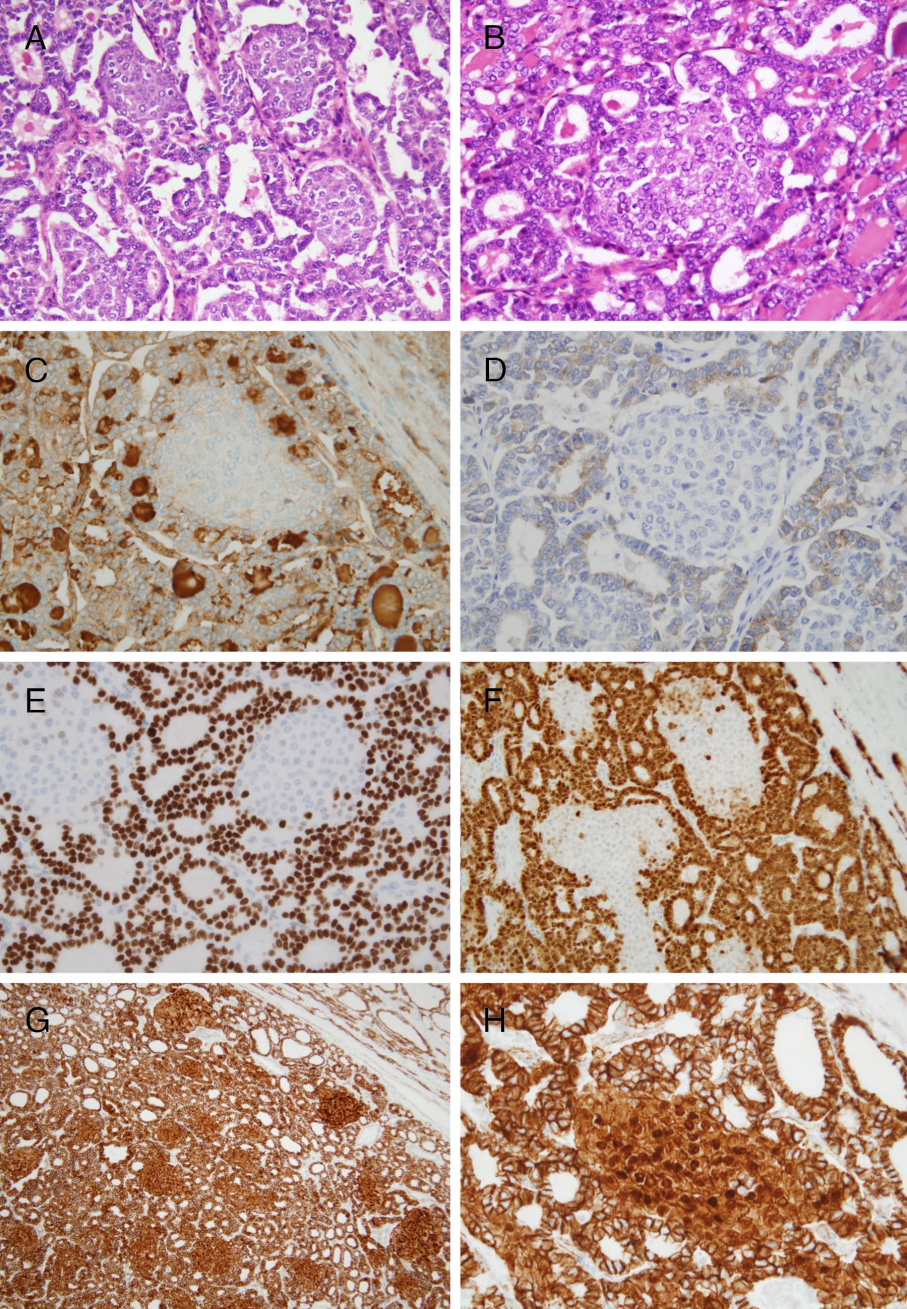
DICER1-related pediatric thyroid neoplasm with follicular and morular growth. In this tumor, the morular structures were scattered among the follicles (**A**, **B**). While follicular structures were positive for thyroglobulin (**C**), thyroperoxidase (**D**), TTF1 (**E**), and PAX8 (**F**), the morular component was negative for thyroglobulin and thyroperoxidase. Only a few isolated cells were found to be positive for TTF1 and PAX8 (**E**, **F**). In contrast to the membranous pattern of the follicular cell component, strong nuclear and cytoplasmic positivity for β-catenin was evident in the morules (**G**, **H**)

The thyroid lobectomy specimen showed a solid encapsulated dominant nodule measuring 15 mm in diameter and multiple non-encapsulated nodules of up to 9 mm with an adenomatous appearance (Fig. 1). Histologically, the main nodule was well demarcated by a thin band of fibrosis and showed a mixed follicular and morular growth pattern (Fig. 2). Areas with a follicular pattern predominated (approximately 60%), with uniform follicles containing colloid, which were somewhat smaller than the follicles in the non-lesional thyroid. The cells of the follicular component were cuboidal and had round nuclei, with frequent chromatin clearing and overlapping but without grooves or nuclear pseudoinclusions (intermediate-type nuclei) (Figs. 2 and 3). The morular component represented approximately 40% of the tumor cell mass, and these morulae (squamoid whorls) were distributed throughout the tumor, occasionally becoming confluent. These morulae were round or oval, lacked keratinization, and displayed some nuclei with peculiar nuclear clearing of the chromatin (Figs. 2 and 3). There were scattered mitotic figures in the follicular component, but no tumor necrosis, infiltration, or vascular invasion was evident.

**Fig. 4:**
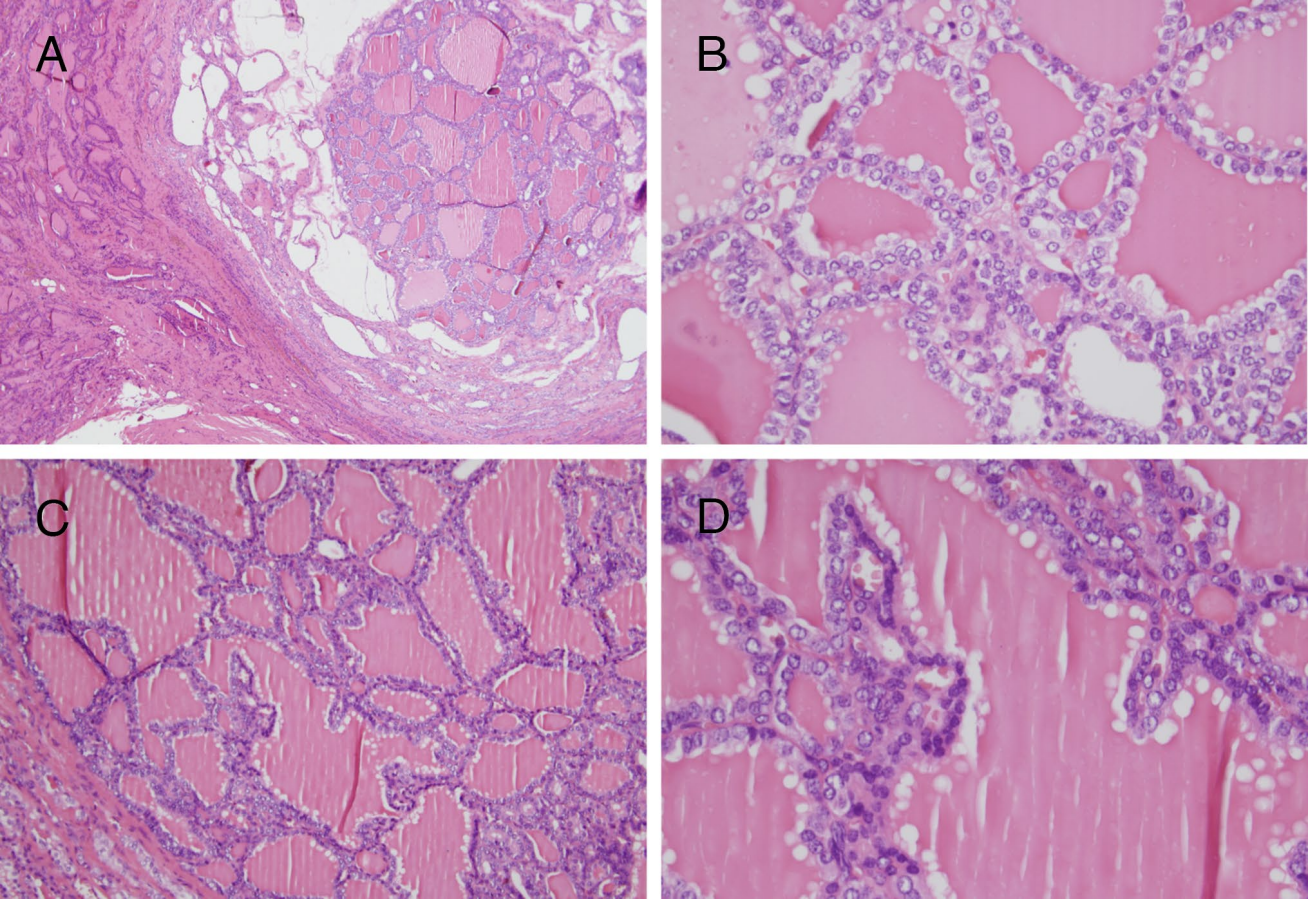
*DICER1*-associated thyroid follicular nodular disease. These two proliferative nodules (**A**, **C**) showed a predominant macrofollicular pattern with some papillary projections. At a higher magnification, nuclear overlapping and clear nuclei could be seen (**B**, **D**)

The remainder of the thyroid parenchyma was occupied by well-circumscribed or partially encapsulated nodules of similar appearance, showing a macrofollicular growth pattern, with colloid-filled follicles and frequent short papillary projections (Figs. 1 and 4). The cells were cuboidal with nuclear characteristics like those of the main encapsulated nodule, including intermediate-type nuclei (Fig. 4B and D). Cystic changes were detected in one of these nodules (Fig. 4A). No significant mitotic activity, necrosis, infiltration, or angioinvasion was detected. No evidence of thyroiditis was found

**Fig. 5:**
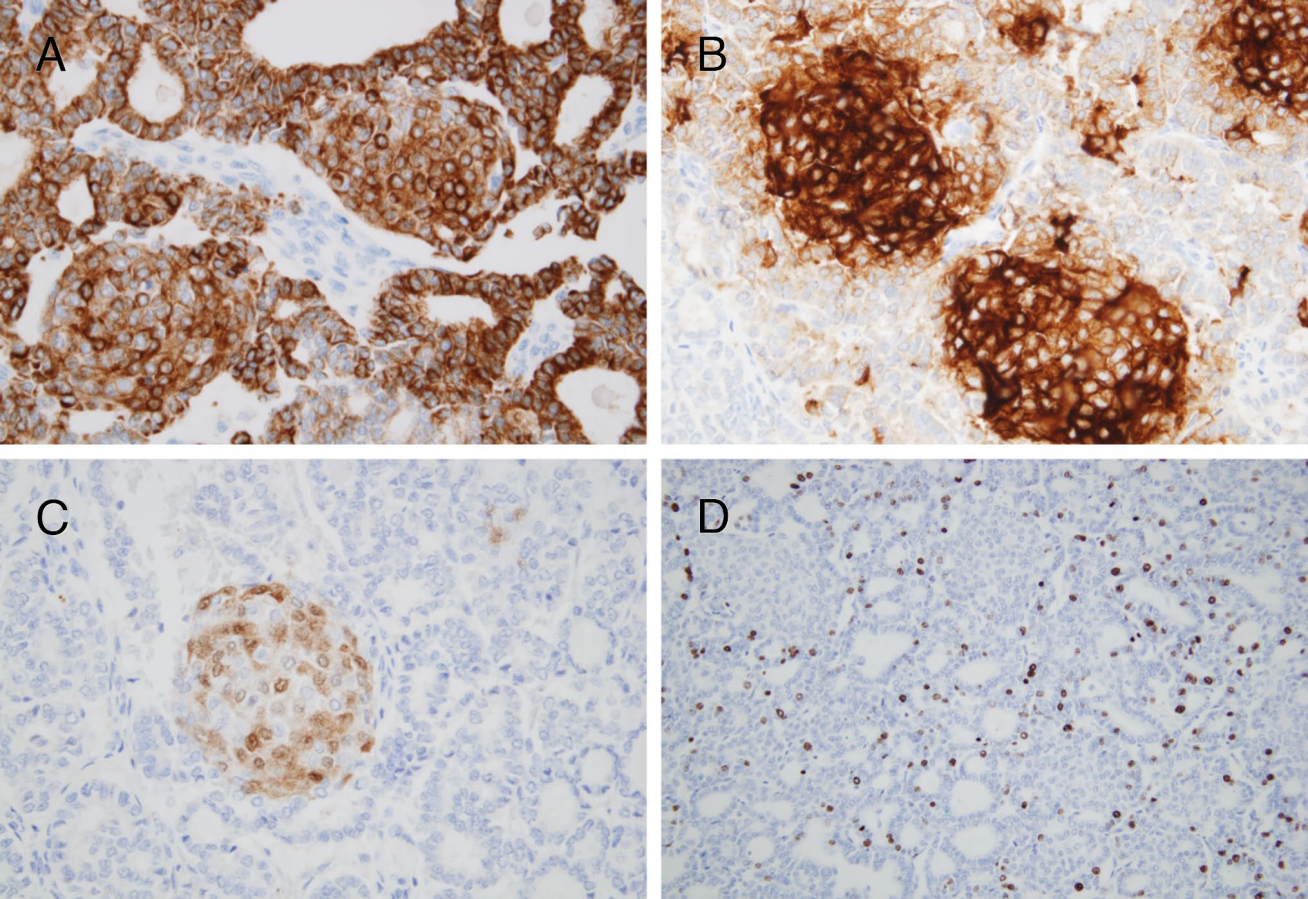
DICER1-related pediatric thyroid neoplasm with follicular and morular growth. Diffuse positivity for CKAE1/AE3 was found both in the follicular and morular components (**A**), but positivity for CD10 (**B**) and CDX2 (**C**) was confined solely to the morular structures. A high Ki67 proliferation index (21%) was restricted to the follicular cell component of the tumor (**D**)

The main results of the immunohistochemical study are shown in Table 1. The follicular component of the encapsulated nodule showed positivity for thyroglobulin, thyroperoxidase, TTF1, PAX8, keratin clones CKAE1/AE3, 34bE12, and CK19 (weak), and vimentin, with a Ki67 proliferation index of 21% (Fig. 3). In contrast, the morular component showed negativity for thyroglobulin, thyroperoxidase, and vimentin, with positivity for TTF1 and PAX8 in isolated cells, diffuse positivity for keratin clones CKAE1/AE3 and CK19, CD10, CDX2, and a Ki67 index of less than 1% (Figs. 3 and 5). While the β-catenin staining pattern of the tumor follicular cells was membranous, the morular component showed a strong nuclear, cytoplasmic staining pattern for β-catenin (Fig. 3G and H). The other nodules present in the thyroid parenchyma showed positivity for thyroglobulin with negativity for calcitonin.

### Genetic Features

The results of the mutational study are shown in Fig. 1. The same *DICER1* (c.4068_4069delGT, p(Tyr1357fs*18) constitutional pathogenic variant was identified in the “normal” thyroid tissue (variant allele frequency [VAF]: 51%), the main nodule (VAF: 50%), and the thyroid adenoma (VAF: 52%). In addition to the constitutional *DICER1* c.4068_4069delGT, p.(Tyr1357fs*18) variant, the oncogenic somatic variant *DICER1* c.5428G > T, p.(Asp1810Tyr) (VAF: 32%) and the oncogenic somatic variant *CTNNB1* c.121A > G, p.(Thr41Ala) (VAF: 23%) were identified in the main nodule. The additional study of one of the macrofollicular adenomas confirmed the constitutional *DICER1* c.4068_4069delGT, p(Tyr1357fs*18) variant, along with two somatic *DICER1* variants c.5126A > G, p.(Asp1709Gly) (VAF: 19%) and c.5429A > T, p.(Asp1810Val) (VAF: 11%). No alterations in the genes *APC*, *AXIN1*, *AXIN2*, *BRAF*, *NRAS*, *HRAS*, *KRAS*, *EIF1AX*, *PTEN*, *TERT*, *PI3KCA*, *KMT2C*, *KMT2D*, *CHEK2*, *ATM*, and *TP53* were detected in any of the samples from the main nodule, thyroid adenoma, or “normal” thyroid. The insufficient quantity and quality of DNA did not allow for specific molecular study of the morular component.

## Discussion

Until now, the spectrum of thyroid alterations associated with constitutional pathogenic variants in *DICER1* has included TFND, differentiated thyroid carcinoma, sometimes combining high-grade areas (tumor-in-tumor pattern) and poorly differentiated thyroid carcinoma (PDTC)  [1, 12]. In *DICER1* syndrome, TFND consists of multiple, bilateral, well-circumscribed nodular proliferations of follicular cells with frequent macrofollicular and/or papillary architecture (so-called papillary hyperplasia or papillary adenoma). Most malignant tumors are well differentiated and have been reported primarily as cases of follicular variant of papillary thyroid carcinoma (PTC) and minimally invasive follicular thyroid carcinoma (FTC) [17–19]. Due to the frequent presence of intermediate-type nuclear characteristics in these lesions [12, 20], however, it is not easy to classify these tumors within the *RAS*-like and *BRAF*-like scheme [21–23]. Therefore, the designation *DICER1*-associated well-differentiated thyroid carcinoma/tumor has been proposed, depending on whether or not they are accompanied by invasive characteristics, respectively [12, 20]. PDTC has also been described in this syndrome using the Turin consensus diagnostic criteria [6, 7, 24]. Interestingly, additional histopathological features such as the detection of multiple areas of thick hyaline ischemic-like stroma (atrophic changes), preferably in the subcapsular region of these tumors or outside the nodules [10–12, 25] as well as the presence of aggregates of ectatic macrofollicles (involutional changes) in the extranodular thyroid [12, 17, 22], may alert the pathologist concerning a somatic and/or germline *DICER1* alteration [9, 12, 13].

Thyroblastoma is an embryonal high-grade triphasic thyroid neoplasm composed of (1) primitive thyroid epithelium (positive for thyroglobulin, TTF1, and PAX8), (2) a blastemal component of small cells positive for SALL-4, and (3) a spindle cell stroma usually positive for alpha-smooth muscle actin, desmin, and myogenin [8, 26]. Thyroblastoma, however, is unrelated to the hereditary *DICER1* syndrome, but is associated with somatic mutations in *DICER1* [26].

In the present case, the thyroid gland showed alterations typically associated with *DICER1* syndrome, including TFND with some nodules showing a predominantly macrofollicular pattern with cystic change and papillary formations in variable proportions. The constitutional *DICER1* mutation was confirmed in histologically normal thyroid tissue, in one of the TNFD nodules and in the main nodule. Other studies have shown different additional somatic mutations of *DICER1* in TFND nodules [12, 27].

To our knowledge, this manuscript describes, for the first time, a thyroid tumor with a peculiar mixed follicular and morular growth pattern that expands the spectrum of thyroid lesions associated with *DICER1* syndrome. In the present tumor, the follicular component of the tumor showed colloid in the follicular lumen surrounded by cells whose nuclei had some characteristics (clearance and overlapping) insufficient for the diagnosis of PTC. These “intermediate-type” nuclear characteristics, following the terminology coined by Sobrinho-Simoes’ group [20], are consistent with those previously described in *DICER1*-associated tumors (see above) and reflect the non-*RAS* and non-*BRAF* pathogenesis of these tumors. In addition to the constitutional *DICER1* p.(Tyr1357fs*18) variant, the somatic *DICER1* p.(Asp1910Tyr) oncogenic variant was also identified in this tumor. The follicular areas of the tumor were positive for thyroglobulin, thyroperoxidase, TTF1, and PAX8, supporting a follicular lineage, while the meaning of the morular structures is intriguing. The morular component, squamoid but not keratinizing, showed positivity for TTF1 and PAX8 in a few isolated peripheral cells, negativity for other follicular lineage markers, and positivity for CD10 and CDX2, as well as intense nuclear and cytoplasmic positivity for β-catenin. Among thyroid tumors, the existence of morulae with a similar immunohistochemical profile has been described in the cribriform morular thyroid carcinoma (CMTC) [14, 28], but similar morulae have also been reported in tumors of other organs such as the low-grade fetal adenocarcinomas of the lung [29], pancreatoblastoma [30], some colorectal adenomas and carcinomas [31, 32], and endometrioid-type endometrial and ovarian carcinomas [33]. All of these belong to the family of tumors containing CD10-positive morulae and share alterations in the WNT/β‐catenin signaling pathway [34]. In addition to the constitutional and somatic *DICER1* oncogenic variants, the somatic *CTNNB1* p.(Thr41Ala) oncogenic variant was also identified in the present tumor, which also justified its histopathogenic relationship with the family of tumors containing CD10-positive morulae.

CMTC is a tumor with a particular histological growth pattern and strong nuclear and cytoplasmic β-catenin staining secondary to constitutive activation of the WNT/β-catenin [35]. CMTC is typically associated with familial adenomatous polyposis but can also occur sporadically [14]. CMTC exhibits an intricate blending of cribriform, follicular, papillary, trabecular, and solid growth patterns, with morular areas. While the follicular/glandular pattern (and all other patterns) of CMTC lacks colloid and is consistently negative for thyroglobulin and PAX8 [14, 35, 36], the follicular component in this present case showed colloid and was positive for these markers. The histogenesis of CMTC is uncertain [28], and a relationship with the thymic/ultimobranchial pouch has been suggested because of the negativity for follicular markers and the immunoreactivity of morulae for CD5 and keratin 5 [36, 37]. Our group, in contrast, has suggested a follicular lineage and has attributed the lack of follicular differentiation of CMTC to the activation of the WNT/β-catenin signaling pathway [14, 35, 38].

WNT/β-catenin signaling is a critical component of intestinal stem cells [39], and it is accepted that the activation of WNT/β‐catenin signaling, through transcription factor CDX2, activates colonic gene expression at high levels [40]. The fact that all CMTC cells show nuclear/cytoplasmic positivity for β-catenin and negativity for thyroglobulin, while the thyroglobulin expression in the present neoplasm is limited to the tumor cells with nuclear/cytoplasmic negativity for β-catenin and vice versa, fits in well with our proposal of a follicular origin for CMTC. While in familial or sporadic CMTC, the permanent activation of the WNT/β-catenin pathway has been explained by alterations in the different components of this pathway [14, 35]; in the present tumor , the restriction of the WNT/β-catenin pathway activation (and nuclear/cytoplasmic positivity for β-catenin) to the morular component distributed throughout the tumor is puzzling. Concurrent with the germline and somatic mutations of *DICER1*, a pathogenic mutation in the *CTNNB1* gene was also confirmed in our present tumor, which would explain the activation of the WNT/β-catenin in the morular structures. Unfortunately, we were unable to obtain sufficient quality DNA from the morulae for a specific molecular analysis. This is a limitation since a more in-depth molecular study of morulae could lead to a better understanding of phenotypic/genetic relationships, particularly in the context of WNT/β-catenin-induced differentiation in DICER1-associated thyroid tumors.

Most low-grade fetal adenocarcinoma/well-differentiated fetal adenocarcinoma (WDFA) of the lung shows nuclear expression of β-catenin and *CTNNB1* somatic mutations [29], but the coexistence of mutations in *CTNNB1* and *DICER1* is uncommon [29]. Analogous to what occurred in this present tumor, however, a case of WDFA of the lung with coexistence of *CTNNB1* and *DICER1* mutations in a 16-year-old girl with a history of TFND, ovarian Sertoli-Leydig cell tumor, and familial *DICER1* syndrome has been described [41].

This DICER1-related pediatric thyroid neoplasm with follicular and morular growth lacks the malignant features typically associated with malignant follicular cell thyroid neoplasms; this tumor is an encapsulated neoplasm with no evidence of invasiveness. Neither is there any evidence of necrosis, and the nuclear atypia is insufficient for a diagnosis of PTC. The presence of mitotic figures and the high Ki67 index in this case appear to reflect the state of the developing young thyroid gland more than the aggressiveness of the tumor [42]. The Ki67 index alone does not appear to impact the prognosis of pediatric thyroid tumors [43], including noninvasive thyroid tumors associated with *DICER1* [23, 44]. For this reason, and in relation to the diagnosis of PDTC, some authors have questioned the use of the Turin consensus criteria in the pediatric population [12, 24]. Finally, although there is no evidence of malignancy, as it is a single tumor, it is not possible to conclusively establish the prognosis of the present tumor.

In conclusion, here, we describe, for the first time, an encapsulated follicular cell thyroid tumor showing a mixed follicular and morular growth pattern, which presented in an 11-year-old girl with follicular nodular disease and a constitutional *DICER1* p.(Tyr1357fs*18) pathogenic variant. The follicular areas were positive for thyroglobulin, while the morular component was positive for CD10 and CDX2. Aberrant (nuclear and cytoplasmic) immunolabeling pattern for β-catenin was limited to the morular structures. In addition to the constitutional *DICER1* p.(Tyr1357fs*18) variant, the somatic *DICER1* p.(Asp1910Tyr) oncogenic variant and the somatic *CTNNB1* p.(Thr41Ala) oncogenic variant were also identified. This peculiar tumor expands the spectrum of *DICER1*-associated thyroid lesions and indirectly establishes a pathogenetic relationship with CMTC.

## Data Availability

No datasets were generated or analysed during the current study.
